# Si-Wu-tang extract stimulates bone formation through PI3K/Akt/NF-κB signaling pathways in osteoblasts

**DOI:** 10.1186/1472-6882-13-277

**Published:** 2013-10-24

**Authors:** Chi-Ming Wu, Po-Chun Chen, Te-Mao Li, Yi-Chin Fong, Chih-Hsin Tang

**Affiliations:** 1 School of Chinese Medicine, China Medical University, Taichung, Taiwan; 2 Department of Life Sciences, National Chung Hsing University, Taichung, Taiwan; 3 Department of Orthopaedics, China Medical University Hospital, Taichung, Taiwan; 4 Graduate Institute of Basic Medical Science, China Medical University, No. 91, Hsueh-Shih Road, Taichung, Taiwan; 5 Department of Pharmacology, School of Medicine, China Medical University, Taichung, Taiwan; 6 Department of Biotechnology, College of Health Science, Asia University, Taichung, Taiwan

**Keywords:** SWT, Osteoblasts, Bone formation, Traditional Chinese medicine

## Abstract

**Background:**

Si-Wu-Tang (SWT), a Traditional Chinese Medicine (TCM) formula, is widely used for the treatment of gynopathies diseases such as menstrual discomfort, climacteric syndrome, dysmenorrhea, and other estrogen-related diseases. Recent studies have shown that SWT can treat primary dysmenorrhea, have anti-pruritic anti-inflammatory effects, and protect against radiation-induced bone marrow damage in an animal model. It has been reported that anti-inflammatory and anti-oxidant agents have the potential to treat osteoporosis by increasing bone formation and/or suppressing bone resorption. However, the effect of SWT on bone cell function has not yet been reported.

**Methods:**

Alkaline phosphatase (ALP), bone morphogenetic proteins (BMP)-2, and osteopontin (OPN) mRNA expression was analyzed by qPCR. The mechanism of action of SWT extract was investigated using western blotting. The *in vivo* anti-osteoporotic effect of SWT extract was assessed in ovariectomized mice.

**Results:**

Here, we report that SWT increases *ALP*, *BMP-2*, and *OPN* expression as well as bone mineralization. In addition, we show that the PI3K, Akt, and NF-κB signaling pathways may be involved in the SWT-mediated increase in gene expression and bone mineralization. Notably, treatment of mice with SWT extract prevented bone loss induced by ovariectomy *in vivo*.

**Conclusion:**

SWT may be used to stimulate bone formation for the treatment of osteoporosis.

## Background

Bone is a mineralized tissue composed of several cell types, which undergoes a continuous renewal and repair process called “bone remodeling”. Bone remodeling is accomplished by bone-forming osteoblasts and bone-resorbing osteoclasts that reside in the bone. The development and differentiation of these 2 cell types are tightly regulated by a number of endogenous substances such as hormones, growth factors, and cytokines [1]. These factors are secreted through the endocrine, paracrine/autocrine, and neurocrine systems, and modulate the balance between bone-forming and bone-resorbing cells in the marrow microenvironment. Osteoporosis results when bone resorption and bone formation are imbalanced and excess bone breakdown exceeds bone building [2]. Bone resorption inhibitors, e.g., bisphosphonates, calcitonin, and estrogen, were designed as therapeutic targets to treat osteoporosis [3]. However, the efficiency of these drugs in improving bone mass is very small, certainly no more than 2% per year [3]. Therefore, teriparatide, an anabolic agent, which stimulates bone formation and corrects characteristic changes in the trabecular microarchitecture in established osteoporosis, is a new approach to treat osteoporosis [4,5]. Bone remodeling is regulated through a balance of bone-forming and bone-resorbing cell activities that together maintain bone mass and mineral homeostasis. New bone formation is mainly controlled by osteoblasts; therefore, agents that act to either increase proliferation of cells of the osteoblastic lineage or induce differentiation of osteoblasts can enhance bone formation [5-7].

The biological mechanism of osteoporosis is still unclear. However, it is likely related to decreased availability or effects of bone growth factors such as bone morphogenetic proteins (BMPs) [8]. BMPs were first discovered as a result of their capacity to induce ectopic bone formation in rodents, and the protein structure of BMPs are similar to the transforming growth factor-β superfamily [9]. BMPs are secreted proteins, which play crucial roles in bone formation and bone cell differentiation through stimulation of alkaline phosphatase (ALP) activity as well as synthesis of proteoglycan, collagen, and osteopontin (OPN) [10]. A previous study showed linkage of osteoporosis to specific polymorphisms in the *BMP-2*, *ALP*, and *OPN* genes, revealing that they are osteoporosis-associated genes [11].

Si-Wu-Tang (SWT), a Traditional Chinese Medicine (TCM) formula, is comprised of a combination of 4 herbs; Paeoniae, Angelicae, Chuanxiong, and Rehmanniae, and is widely used for the treatment of women’s diseases such as cutaneous pruritus and chronic inflammation, and other diseases. Modern pharmacological studies have shown that SWT extract has anti-pruritic [12] and anti-inflammatory effects [12], and protects against radiation-induced bone marrow damage in an animal model [13,14]. Previous studies have shown that anti-inflammatory and anti-oxidant agents have the potential to treat osteoporosis by increasing bone formation and/or suppressing bone resorption [15,16]. However, the effect of SWT on bone cell function has not yet been reported. In the current study, we report that SWT extract increases *ALP*, *BMP-2*, and *OPN* expression and bone mineralization. Furthermore, we show that the phosphatidylinositol 3-kinase (PI3K), Akt, and NF-κB signaling pathways are involved in the SWT-mediated increase in gene expression and bone mineralization. Finally, treatment of mice with SWT extract prevented bone loss induced by ovariectomy *in vivo*. Our data, therefore, suggest that SWT may be used to stimulate bone formation for the treatment of osteoporosis.

## Methods

### SWT extract and materials

SWT extract was kindly provided by Timing Pharmaceutical Company (New Taipei City, Taiwan). The extraction and isolation of SWT were performed as previously described [17]. Rabbit polyclonal antibodies for BMP-2, OPN, p-p85 (Tyr^458^), p85, p-Akt (Ser^473^), Akt, p-p65 (Ser^536^), and p65 were purchased from Santa Cruz Biotechnology (Santa Cruz, CA, USA). The osteopontin BMP-2 ELISA kit was purchased from Biosource Technology (Nivelles, Belgium). The C-terminal telopeptides of type-I collagen ELISA kit was obtained from Cross Laps (Herlev, Denmark). p85 and Akt siRNAs were purchased from Santa Cruz Biotechnology. All other reagents were obtained from Sigma-Aldrich (St. Louis, MO, USA).

### Cell culture

The murine osteoblast cell line MC3T3-E1 was purchased from American Type Culture Collection (ATCC; Rockville, MD, USA). Cells were cultured in 5% CO_2_ with α-MEM supplemented with 20 mM HEPES and 10% heat-inactivated fetal calf serum, 2 mM glutamine, penicillin (100 units/mL), and streptomycin (100 μg/mL).

### Measurement of mineralized nodule formation

Levels of mineralized nodule formation were evaluated as previously described [18,19]. Briefly, osteoblasts were cultured in medium containing vitamin C (50 μg/mL) and β-glycerophosphate (10 mM) for 2 wks, and the medium was changed every 3 d. After incubation with SWT extract for 12 d, cells were washed twice with 20 mM Tris-buffered saline containing 0.15 M NaCl (pH 7.4), fixed in ice-cold 75% (v/v) ethanol for 30 min, and air-dried. Calcium deposition was determined using alizarin red-S staining. Briefly, ethanol-fixed cells and matrix were stained for 1 h with 40 mM alizarin red-S (pH 4.2) and rinsed extensively with water. The bound stain was eluted with 10% (w/v) cetylpyridinium chloride, and alizarin red-S in the samples was quantified by measuring absorbance at 550 nm and comparing to a standard curve. One mole of alizarin red-S selectively binds approximately 2 moles of calcium.

### Quantitative real time PCR

Total RNA was extracted from osteoblasts using a TRIzol kit (MDBio Inc., Taipei, Taiwan). Reverse transcription was performed using 2 μg of total RNA and oligo(dT) primers [20,21]. Quantitative real-time PCR (qPCR) was carried out using TaqMan® One-Step PCR Master Mix (Applied Biosystems, Carlsbad, CA, USA). cDNA (100 ng) was added to a 25-μL reaction containing sequence-specific primers and Taqman® probes. All target gene primers and probes were purchased commercially, including β-actin as an internal control (Applied Biosystems). qPCR assays were carried out in triplicate on a StepOnePlus sequence detection system (Applied Biosystems). The cycling conditions were as follows: 10-min polymerase activation at 95°C followed by 40 cycles of 95°C for 15 s and 60°C for 60 s. The threshold was set above the non-template control background and within the linear phase of target gene amplification to calculate the cycle number at which the transcript was detected (denoted C_T_).

### Cell viability

Cell viability was determined by 3-[4,5-dimethylthiazol-2-yl]-2,5-diphenyltetrazoliumbromide (MTT) assay. After treatment with SWT extract for 2 days, cultures were washed with PBS. MTT (0.5 mg/ml) was then added to each well and the mixture was incubated for 2 h at 37°C. Culture medium was then replaced with equal volume of DMSO to dissolve formazan crystals. After shaking at room temperature for 10 min, absorbance of each well was determined at 550 nm using a microplate reader (Bio-Tek, Winooski, VT).

### Western blot analysis

Cell lysates were prepared as described previously [22]. Proteins were resolved by SDS-PAGE and transferred to Immobilon polyvinyldifluoride membranes (Millipore, Billerica, MA, USA). The blots were blocked with 4% bovine serum albumin for 1 h at room temperature, and then probed with rabbit anti-human antibodies against p85, p-p85, p-Akt, Akt, p65, or p-p65 (1:1000) for 1 h at room temperature. After 3 washes, the blots were incubated with peroxidase-conjugated donkey anti-rabbit secondary antibody (1:1000) for 1 h at room temperature. The blots were visualized by enhanced chemiluminescence using X-OMAT LS film (Eastman Kodak, Rochester, NY).

### Ovariectomy-induced osteoporosis

Female ICR mice (4 wks old; 22–28 g) were used for this study. Mice were ovariectomized bilaterally under trichloroacetaldehyde (100 mg/kg) anesthesia and control mice were sham-operated (Sham) for comparison. Bone mineral density and bone mineral content were measured after oral administration of various concentrations of SWT extracts every 2 d for 4 wks. Total body bone mineral density and bone mineral content were determined by a dual-energy X-ray absorptiometer (DEXA; XR-26; Norland, Fort Atkinson, WI) using a mode for small subjects as described previously [19,23]. All protocols complied with institutional guidelines and were approved by the Animal Care Committee of China Medical University.

### Statistical analysis

Statistical analysis was performed using Prism 4.01 software (GraphPad Software Inc., San Diego, CA, USA). The values given are means ± standard errors of the mean (SEM). Statistical analyses between 2 samples were performed using the Student’s *t*-test. Statistical comparisons of more than 2 groups were performed using 1-way analysis of variance with Bonferroni’s *post-hoc* test. In all cases, *p* < 0.05 was considered significant.

## Results

### SWT extract increases bone mineralization by osteoblasts

**Figure 1:**
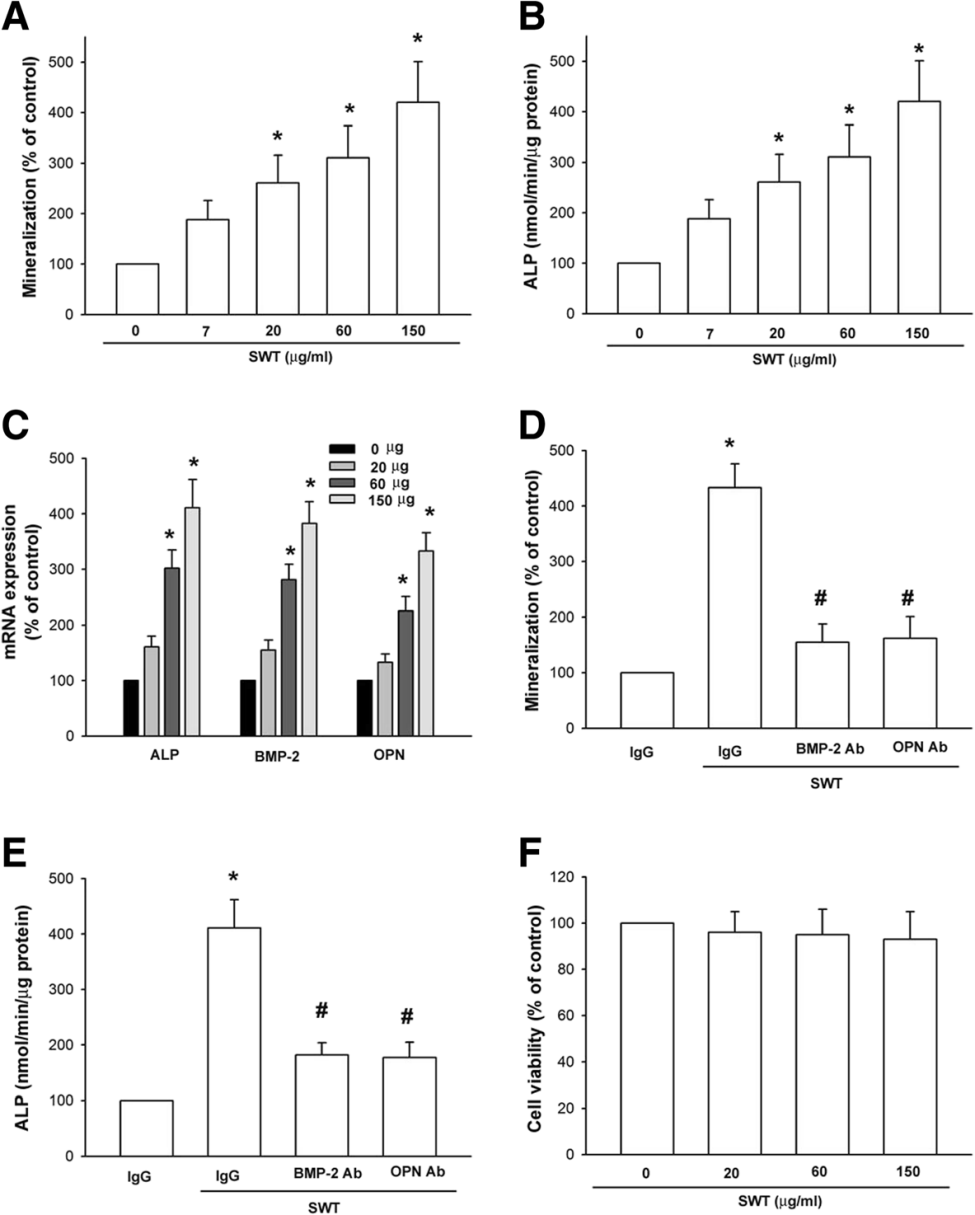
**SWT extract increases bone mineralization in cultured osteoblasts. (A)** Osteoblasts were seeded in 24-well plates and cultured for 2 wks in medium containing vitamin C (50 μg/mL) and β-glycerophosphate (10 mM). The cells were concomitantly treated with SWT extract. At the end of the experiment, cultures were fixed in 75% ethanol, and mineralized nodule formation was assessed by alizarin red-S staining. The bound stain was eluted with a solution of 10% cetylpyridinium chloride and quantified using a microtiter plate reader. **(B)** Cells were incubated with SWT extract for 72 h, and ALP was measured with an ALP activity assay kit. **(C)** Cells were incubated with SWT extract for 24 h, and *ALP*, *BMP-2*, and *OPN* mRNA expression was measured by qPCR. **(D)** Cells were incubated with SWT extract (150 μg/mL) plus BMP-2, OPN, or IgG (negative control) neutralizing antibodies. Mineralized nodule formation was assessed by alizarin red-S staining. **(E)** Cells were incubated with SWT extract (150 μg/mL) plus BMP-2, OPN, or IgG (negative control) neutralizing antibodies for 72 h, and ALP was measured with an ALP activity assay kit. **(F)** Cells were incubated with SWT extract, the cell viability was examined by MTT assay. Results are expressed as mean ± SEM *, *p* < 0.05 compared to the control group; #, *p* < 0.05 compared to the SWT extract-treated group.

In this study, we investigated the role of SWT in osteoblast differentiation. The formation of mineralized nodules is a marker of osteoblast maturation. Alizarin red-S staining showed that mineralized nodules formed when osteoblasts were cultured for 2 wks in medium containing vitamin C (50 μg/mL) and β-glycerophosphate (10 mM), and this increased in a concentration-dependent manner with the addition of SWT (Figure 1A). Differentiated osteoblasts exhibit elevated ALP activity, which correlates with high levels of enzyme expression [18,24]. Therefore, we assessed the effects of SWT on osteoblast ALP activity, and our results showed that treatment with SWT extract for 72 h significantly increased ALP activity (Figure 1B). It is a general view that BMP-2, ALP, and OPN have crucial roles in osteoblast differentiation. We tested whether SWT extract mediates its effects on osteoblast differentiation by regulation of the expression of *BMP-2*, *ALP*, and *OPN*. Treatment of cells with SWT extract increased the mRNA expression of *ALP*, *BMP-2*, and *OPN* in a concentration-dependent manner (Figure 1C). To investigate whether the induction of BMP-2 and OPN expression is critical for SWT-promoted osteoblast differentiation, we assessed the inhibitory effects of a neutralizing antibody against BMP-2 and OPN. Our data showed that SWT-induced bone nodule formation and *ALP* mRNA expression was significantly decreased after treatment with the neutralizing antibody (Figure 1D and E). However, SWT did not affect cell viability in osteoblasts (Figure 1F). These results demonstrated that SWT extract induced differentiation of osteoblasts by upregulating *BMP-2*, *ALP*, and *OPN* expression.

### SWT extract increases bone nodule formation through the PI3K/Akt pathway

**Figure 2:**
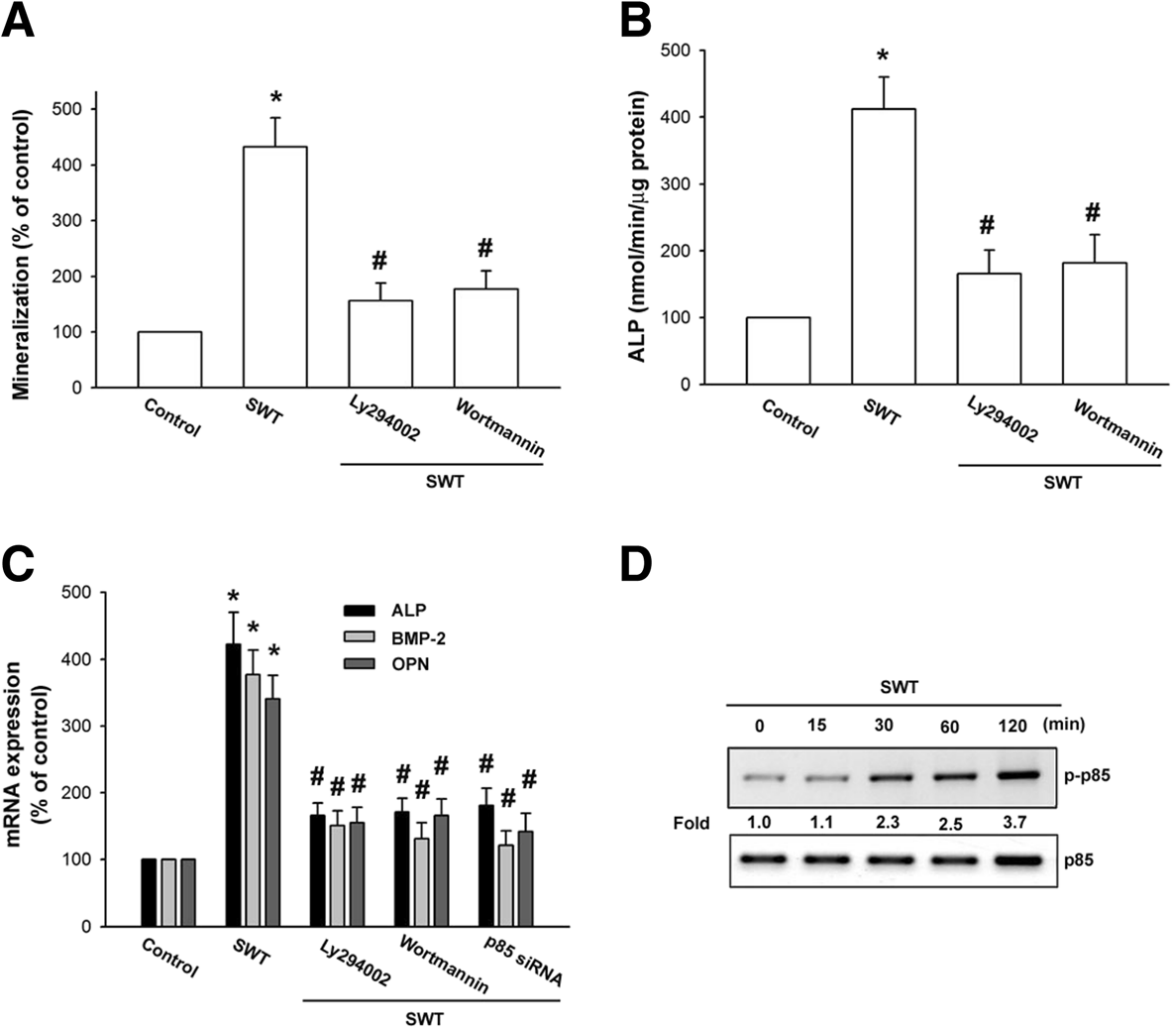
**Involvement of PI3K in SWT extract-induced bone mineralization in osteoblasts. (A)** Cells were seeded in 24-well plates and cultured for 2 wks in medium containing vitamin C (50 μg/mL) and β-glycerophosphate (10 mM). The cells were concomitantly treated with SWT extract (150 μg/mL) plus Ly294002 or wortmannin. Mineralized nodule formation was assessed by alizarin red-S staining. **(B)** Cells were incubated with SWT extract (150 μg/mL) plus Ly294002 or wortmannin for 72 h, and ALP was measured with an ALP activity assay kit. **(C)** Cells were pretreated with Ly294002 and wortmannin for 30 min or transfected with p85 siRNA for 24 h followed by stimulation with SWT extract for 24 h, and *ALP*, *BMP-2*, and *OPN* mRNA expression was measured by qPCR. **(D)** Cells were treated with SWT extract for the indicated time intervals, and p-p85 phosphorylation was examined by western blotting. *, *p* < 0.05 compared to the control group; #, *p* < 0.05 compared to the SWT extract-treated group.

**Figure 3:**
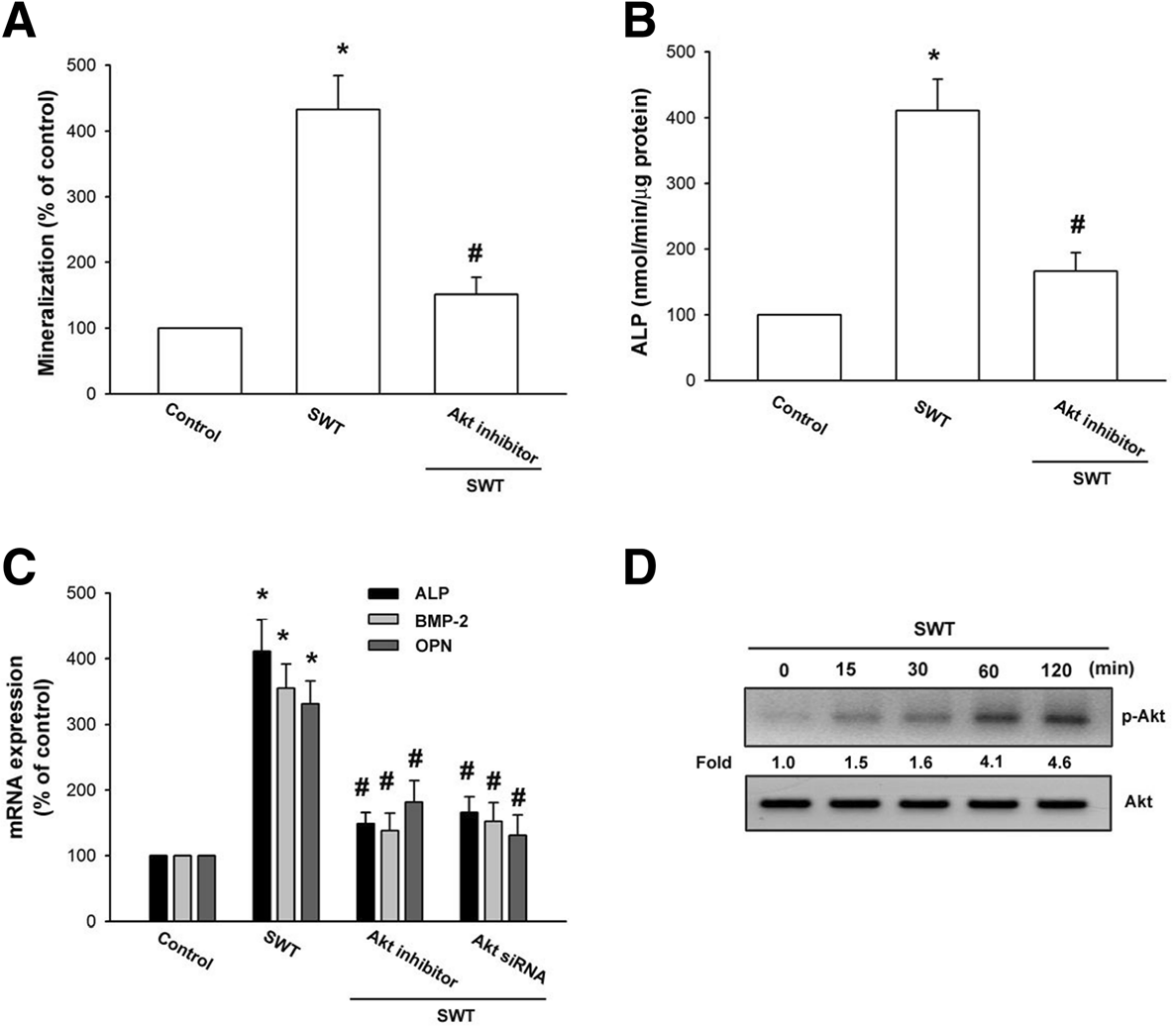
**Involvement of Akt in SWT extract-induced bone mineralization in osteoblasts. (A)** Cells were seeded in 24-well plates and cultured for 2 wks in medium containing vitamin C (50 μg/mL) and β-glycerophosphate (10 mM). The cells were concomitantly treated with SWT extract (150 μg/mL) plus Akt inhibitor. Mineralized nodule formation was assessed by alizarin red-S staining. **(B)** Cells were incubated with SWT extract (150 μg/mL) plus Akt inhibitor for 72 h, and ALP was measured with an ALP activity assay kit. **(C)** Cells were pretreated with Akt inhibitor for 30 min or transfected with Akt siRNA for 24 h followed by stimulation with SWT extract for 24 h, and *ALP*, *BMP-2*, and *OPN* mRNA expression was measured by qPCR. **(D)** Cells were treated with SWT extract for the indicated time intervals, and p-Akt expression was examined by western blotting. *, *p* < 0.05 compared to the control group; #, *p* < 0.05 compared to the SWT extract-treated group.

It has been reported that PI3K and Akt play an important role in bone formation [25,26]. We next examined whether these signaling pathways are involved in SWT extract-induced bone mineralization. The osteoblasts were pretreated with a PI3K inhibitor (Ly294002 and wortmannin) or an Akt inhibitor for 30 min and then incubated with SWT extract for 24 h. Pretreatment of cells with these pathway inhibitors reduced SWT extract-induced bone mineralization (Figures 2A and 3A). The inhibitors also decreased ALP activity that was upregulated by SWT extract (Figures 2B and 3B). Furthermore, pretreatment with the inhibitors or transfection of cells with p85 and Akt siRNA blocked SWT extract-induced *ALP*, *BMP-2*, and *OPN* mRNA expression (Figures 2C and 3C). Next, we directly examined p85 and Akt activation after SWT extract treatment. Incubation of cells with SWT extract induced p85 and Akt phosphorylation (Figures 2D and 3D). Therefore, these results indicate that the PI3K and Akt pathways are involved in SWT extract-induced bone formation in osteoblasts.

### SWT extract increases bone nodule formation through the NF-κB pathway

**Figure 4:**
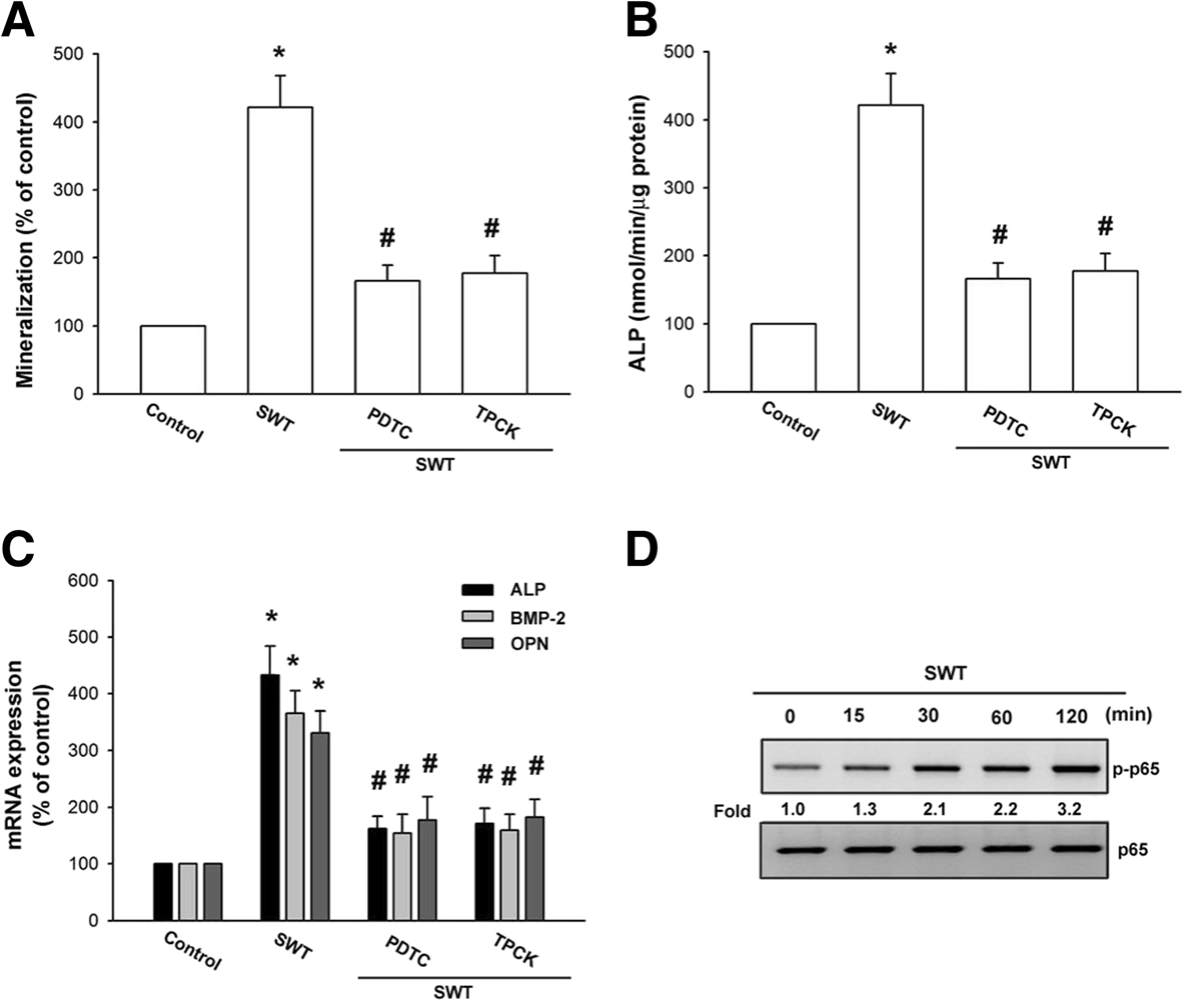
**Involvement of NF-κB in SWT extract-induced bone mineralization in osteoblasts. (A)** Cells were seeded in 24-well plates and cultured for 2 wks in medium containing vitamin C (50 μg/mL) and β-glycerophosphate (10 mM). The cells were concomitantly treated with SWT extract (150 μg/mL) plus PDTC or TPCK. Mineralized nodule formation was assessed by alizarin red-S staining. **(B)** Cells were incubated with SWT extract (150 μg/mL) plus PDTC or TPCK for 72 h, and ALP was measured with an ALP activity assay kit. **(C)** Cells were pretreated with PDTC or TPCK for 30 min followed by stimulation with SWT extract for 24 h, and *ALP*, *BMP-2*, and *OPN* mRNA expression was measured by qPCR. **(D)** Cells were treated with SWT extract for the indicated time intervals, and p-p65 phosphorylation was examined by western blotting. *, *p* < 0.05 compared to the control group; #, *p* < 0.05 compared to the SWT extract-treated group.

As mentioned above, NF-κB activation is necessary for bone formation [27,28]. We next pretreated osteoblasts with NF-κB inhibitors (PDTC and TPCK) to determine whether NF-κB activation is involved in SWT extract-induced bone mineralization. The results showed that pretreatment of osteoblasts with PDTC or TPCK inhibited SWT extract-induced bone nodule formation; ALP activity; and *ALP*, *BMP-2*, and *OPN* mRNA expression (Figure 4A–C). NF-κB activation depends on phosphorylation of the NF-κB p65 subunit [29]. Our results indicate that SWT extract increased p65 phosphorylation in osteoblasts (Figure 4D), showing that NF-κB activation is crucial for SWT extract-induced expression of *ALP*, *BMP-2*, and *OPN*, as well as bone nodule formation.

### Inhibition of bone loss by SWT extract in ovariectomized mice

**Figure 5:**
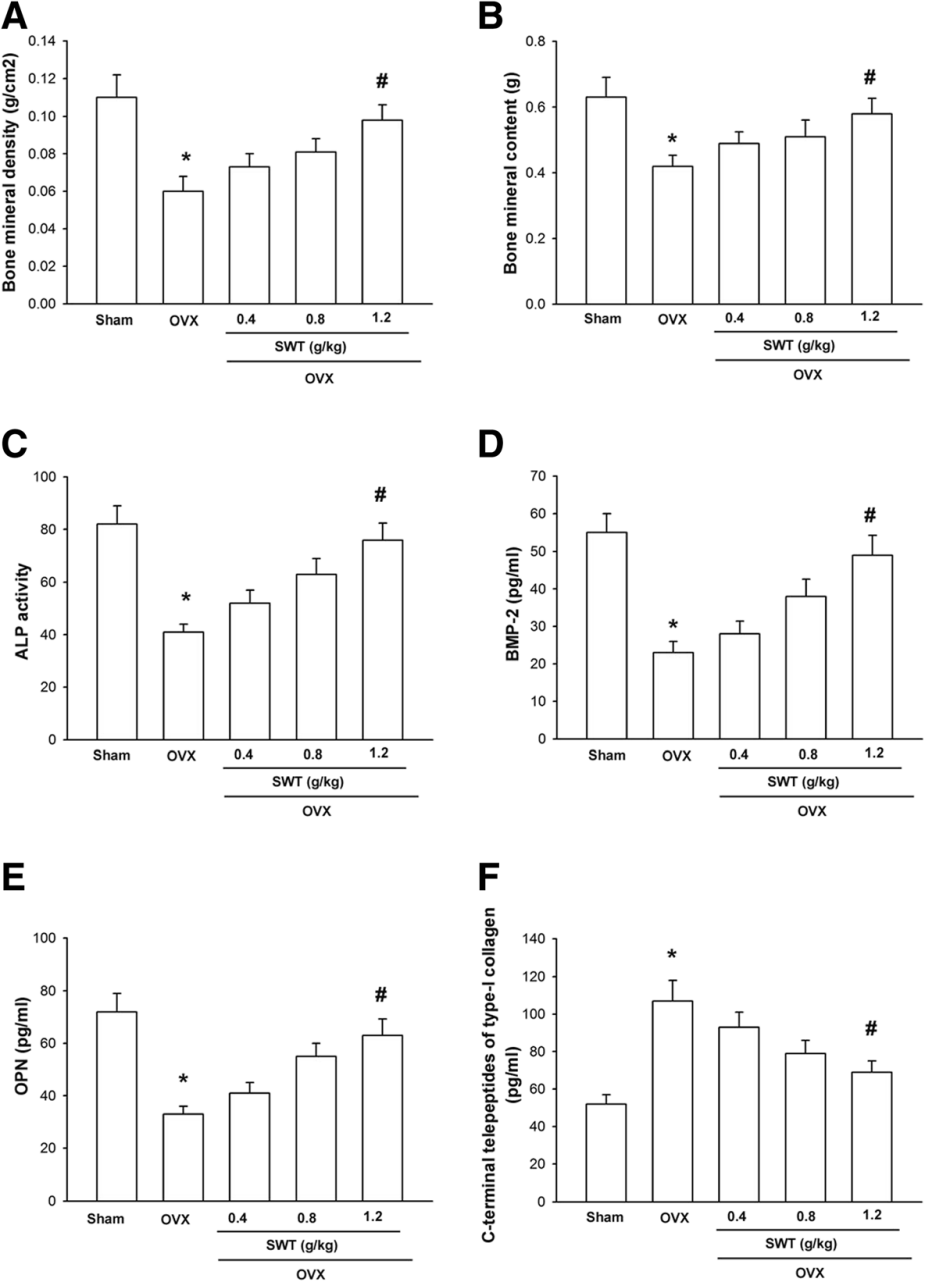
**Inhibition of ovariectomy-induced decrease in bone mineral density and bone mineral content by SWT extract. (A)** Female ICR mice were given a sham operation or were ovariectomized. Mice that underwent ovariectomy were treated with the indicated concentrations of SWT extract by oral feeding. Total body bone mineral density **(A)**, bone mineral content **(B)**, serum ALP activity **(C)**, serum BMP-2 **(D)**, serum OPN **(E)**, and serum C-terminal telopeptides of type I collagen **(F)**, were determined 4 wks after surgery. Data are presented as mean ± SEM (n = 8–10 mice/group). *, *p* < 0.05 compared to sham; #, *p* < 0.05 compared to the ovariectomized group.

To assess the effects of SWT extract on bone loss, an osteoporosis model was used, with female ovariectomized mice. As expected, ovariectomized mice displayed decreased total body bone mineral density and bone mineral content (Figure 5A and B). However, treatment with SWT extract for 4 wks reversed the loss in bone mineral density and bone mineral content in a dose-dependent manner (Figure 5A and B). Blood ALP concentration is correlated with osteoblastic activity [30], and we found that SWT extract inhibited the decrease in serum ALP activity induced by ovariectomy (Figure 5C). SWT extract also increased the levels of BMP-2 and OPN, markers of bone formation, and reduced the level of C-terminal telopeptides of type I collagen, a marker of bone resorption (Figure 5D–F). These findings open a new avenue for SWT extract in the prevention of bone loss *in vivo*.

## Discussion

Si-Wu-Tang, a TCM formula, is widely used in traditional medicine for various therapeutics, including women’s diseases, chronic inflammation, and other diseases because of its anti-pruritic and anti-inflammatory effects [12]. In this study, we showed that SWT extract induced bone mineralization in cultured osteoblasts. In addition, we found that SWT extract increased the expression levels of *ALP*, *BMP-2*, and *OPN*, which requires the activation of PI3K, Akt, and NF-κB signaling pathways. SWT is comprised of a combination of 4 herbs; Paeoniae, Angelicae, Chuanxiong, and Rehmanniae. On the other hand, the major bioactive components in these 4 herbs include phenolics, phthalides, alkaloids, terpene glycosides, and iridoid glycosides. In the current study, we used SWT extract to examine the role SWT in bone formation. However, we did not extract and examine the role of single compound in SWT. Therefore, the next step is to disclose which compound is most important in SWT extract.

Bone is a complex tissue composed of several cell types that are continuously undergoing a process of renewal and repair [31]. Osteoporosis results from an imbalance between bone resorption and bone formation, where bone breakdown overrides bone formation [31]. We took advantage of the ovariectomized mouse model to examine the anti-osteoporotic effects of SWT extract. The results showed that ovariectomized mice had reduced total body bone mineral density and bone mineral content, and this was reversed by treatment with SWT extract. SWT extract also increased serum levels of the osteogenic markers ALP, BMP-2, and OPN. Therefore, SWT is a novel bone formation agent, which prevents bone loss by ovariectomy *in vivo*.

The molecular mechanisms underlying osteoporosis are not yet entirely clear. However, they are likely correlated with decreased availability or activity of bone growth factors, including ALP, BMP-2, and OPN. These 3 factors play important roles in the process of bone formation and remodeling [23], and it has been well discussed that stimulation of osteoblast cell differentiation is characterized mainly by increased expression of ALP, BMP-2, and OPN [32]. In this study, we found that SWT extract increased ALP, BMP-2, and OPN expression and enhanced bone mineralization. Therefore, SWT extract mediates bone formation by upregulating the expression of *ALP*, *BMP-2*, and *OPN*.

Previous studies have reported that PI3K and Akt play important roles in bone formation [25,26]. Phosphorylation of the p85 subunit is required for activation of the p110 catalytic subunit of PI3K [33]. Here, we showed that SWT extract induced PI3K and Akt phosphorylation, and that pretreatment with inhibitors of these signal proteins antagonized the SWT extract-mediated potentiation of bone mineralization, revealing that PI3K and Akt activation play crucial roles in SWT extract-induced bone formation by osteoblasts. Moreover, inhibitors and siRNA of PI3K and Akt reduced SWT extract-dependent enhancement of *ALP*, *BMP-2*, and *OPN* expression. These results suggest that activation of the PI3K and Akt pathways are required for increased *ALP*, *BMP-2*, and *OPN* expression and maturation by SWT extract in osteoblasts. It has been reported that p38 is involved in the regulation of ALP expression during the differentiation of osteoblastic cells [34]; similarly ERK1/2 is important for the proliferation and differentiation of osteoblasts [35]. JNK is involved in osteoblast formation [36]. However, we did not examine the role of MAPKs (p38, JNK, and ERK) in SWT extract-mediated bone formation in current study. Whether MAPKs are involved in SWT extract-induced bone formation needs further examination.

NF-κB has been shown to control osteoblast function in bone [37]. The results of our study indicate that NF-κB activation contributes to SWT extract-induced bone mineralization and *ALP*, *BMP-2*, and *OPN* expression in cultured osteoblasts, and that inhibitors of the NF-κB signaling pathway, including PDTC or TPCK, inhibited SWT extract-induced bone mineralization and the expression of *ALP*, *BMP-2*, and *OPN*. Phosphorylation at Ser^536^ of p65 is crucial for p65 transactivation [38]. The results of this study showed that SWT extract increased the phosphorylation of p65. Taken together, these results suggest that NF-κB activation is required for SWT extract-induced bone formation in cultured osteoblasts.

## Conclusion

Our present study indicated that SWT extract induces osteoblast differentiation and maturation. SWT extract also increased *ALP*, *BMP-2*, and *OPN* expression, and bone mineralization. SWT extract-mediated bone formation and the expression of *ALP*, *BMP-2*, and *OPN* were mediated through PI3K, Akt, and NF-κB signaling pathways. Furthermore, SWT extract reversed *in vivo* bone loss induced by ovariectomy. In conclusion, SWT may be beneficial in stimulating bone formation for the treatment of osteoporotic diseases.

## Competing interests

The authors have no financial or personal relationships that could inappropriately influence this research.

## Authors’ contributions

Conceived and designed the experiments: CM Wu and CH Tang. Performed the experiments: CM Wu, PC Chen, TM Li, and YC Fong. Wrote the paper: CM Wu, PC Chen, and CH Tang. All authors read and approved the final manuscript.

## Pre-publication history

The pre-publication history for this paper can be accessed here:

http://www.biomedcentral.com/1472-6882/13/277/prepub
